# Design and Prototyping of an Underactuated Hand Exoskeleton With Fingers Coupled by a Gear-Based Differential

**DOI:** 10.3389/frobt.2022.862340

**Published:** 2022-03-29

**Authors:** Mihai Dragusanu, Danilo Troisi, Alberto Villani, Domenico Prattichizzo, Monica Malvezzi

**Affiliations:** ^1^ Department of Information Engineering and Mathematics, University of Siena, Siena, Italy; ^2^ Information Engineering Department, University of Pisa, Pisa, Italy; ^3^ Department of Advanced Robotics, Istituto Italiano di Tecnologia, Genova, Italy

**Keywords:** wearable device, exoskeleton, differential mechanism, prototyping, rehabilitation

## Abstract

Exoskeletons and more in general wearable mechatronic devices represent a promising opportunity for rehabilitation and assistance to people presenting with temporary and/or permanent diseases. However, there are still some limits in the diffusion of robotic technologies for neuro-rehabilitation, notwithstanding their technological developments and evidence of clinical effectiveness. One of the main bottlenecks that constrain the complexity, weight, and costs of exoskeletons is represented by the actuators. This problem is particularly evident in devices designed for the upper limb, and in particular for the hand, in which dimension limits and kinematics complexity are particularly challenging. This study presents the design and prototyping of a hand finger exoskeleton. In particular, we focus on the design of a gear-based differential mechanism aimed at coupling the motion of two adjacent fingers and limiting the complexity and costs of the system. The exoskeleton is able to actuate the flexion/extension motion of the fingers and apply bidirectional forces, that is, it is able to both open and close the fingers. The kinematic structure of the finger actuation system has the peculiarity to present three DoFs when the exoskeleton is not worn and one DoF when it is worn, allowing better adaptability and higher wearability. The design of the gear-based differential is inspired by the mechanism widely used in the automotive field; it allows actuating two fingers with one actuator only, keeping their movements independent.

## 1 Introduction

Nowadays, the innovation in the rehabilitation processes and in assistive supports is guided by a twofold thrust. First, the overall social impact of chronic diseases related to the musculoskeletal and nervous system is becoming relevant because the mean age of the population is increasing owing to a better quality of life. Indeed, recent statistics published by the World Health Organization (WHO) shows that nearly one billion people worldwide are suffering due to musculoskeletal and neurological diseases (World Health Organization, 2016). According to such statistics (World Health Organization, 2021), in 2019, people were living more than 6 years longer than in 2000, but on average, only five of those additional years were lived in good health. Second, the spreading of technology in everyone’s day-to-day life is becoming an important tool for preserving and guaranteeing a high-quality life also in the presence of temporary and/or permanent diseases. Technology advancements in the medical and assistive field constitute an important resource for people with disabilities, helping them in moving, performing manual tasks, communication and learning, providing autonomy in their activities of daily living (ADLs), and globally in the whole process of integration. Innovations in technology are progressively changing the rehabilitation environment. The robot-mediated therapies are an emerging and promising field that incorporates robotics with neuroscience and rehabilitation to define new systems for supporting individuals with neurological diseases (Peretti et al., 2017; Díaz et al., 2018; Martinez-Martin and Cazorla, 2019).

With the COVID-19 pandemic, that dramatically modified our habits, the request of innovative technological resources for rehabilitation has been significantly improved in order to be delivered remotely (Anthonius Lim and Pranata, 2020). Therefore, in the immediate future, telerehabilitation could further spread and become more common, even necessary. Moreover, there are evident advantages in distance rehabilitation, whether synchronous (real-time) or asynchronous (store-and-forward). In fact, the availability of tools for autonomously performing physiotherapy exercises increases their intensity and efficiency, provides supplementary information about results and progress, reduces physiotherapist efforts and the need for their physical presence during exercise sessions, and encourages autonomy and independence in people with disabilities. The research in this field is still in progress but suggests some health benefits in the use of exoskeletons in rehabilitation and assistive tasks, including improvements in gait function, body composition, aerobic capacity, bone density, and quality of life (Gorgey et al., 2019).

Concerning the hand, finger flexion and extension exercises, according to the disease of the subject, have an important effect on the recovery (Lum et al., 2002). Moreover, in general, the execution of repetitive movements of the hand and wrist with a controllable intensity is an important part of the rehabilitation process (Dragusanu et al., 2020a). In this context, we focus on the upper limbs and, in particular, on the hand and wrist. They play an important role in all ADLs, and therefore significant research effort is focused on developing exoskeleton devices designed to retrain these parts of the human body (Hesse et al., 2003; Gopura et al., 2016; Rehmat et al., 2018). The aim of hand exoskeletons is to emulate the physical effort of the therapist by producing the same movements able to maintain the physical abilities of the patient. Nonetheless, the presence of the physiotherapist is usually required and covers a supervisor role. The use of hand exoskeletons can be beneficial as it requires a smaller workforce, allows a more lasting and more intense therapy, and reduces the need for long physical contacts and close personal distance between the therapist and the patient.

There are different types of hand exoskeletons that have been developed on the basis of different criteria, that is, size, weight, degrees of freedom (DoF), flexibility, wearability, modularity, and actuation mechanism (Moreno-SanJuan et al., 2021; du Plessis et al., 2021; Shahid et al., 2018). In the studies by Krebs et al., (2007; Gupta et al., (2008; Bartlett et al., (2015; Dragusanu et al., (2020a), the researchers focused on the exoskeleton for the wrist. Hand exoskeleton design is a still open and challenging engineering and research topic since the human hand has a quite complex kinematic structure. Broadly speaking, a fully actuated solution would require an actuator for each DoF, so, in theory, we would need four actuators for each actuated finger (Malvezzi et al., 2020). Rahman and Al-Jumaily (2012) present a fifteen degree of freedom (DoFs) hand exoskeleton based on compliant mechanisms that flex and extend the fingers by using bilateral movement training. In the study by Zhang et al., (2014) a hand exoskeleton for rehabilitation purposes due to injuries is shown. It is actuated by using a Bowden cable setup driven by DC motors, and it can be adjusted to various hand sizes owing to the rack and pinion slide mechanism. The same cable mechanisms are used by Randazzo et al. (2017) and by Marconi et al. (2019). In the study by Popov et al., (2016), the authors present a glove-type exoskeleton that actuates three fingers (excluding the little finger) and the thumb by using tendon cables routed in a glove, while a different design approach is exploited for the hand exoskeletons based on the rigid mechanical structure. These kinds of devices use motors directly connected to the structure that transmit the motion to the required joints. The most popular devices in this area are the ones based on the remote center of rotation (Wang et al., 2018), 4-bar linkage mechanisms (Bianchi et al., 2018), base-to-distal devices with mechanical links connected in series (Jo et al., 2019), and matched axis mechanical structure (Bataller et al., 2016).

**FIGURE 1:**
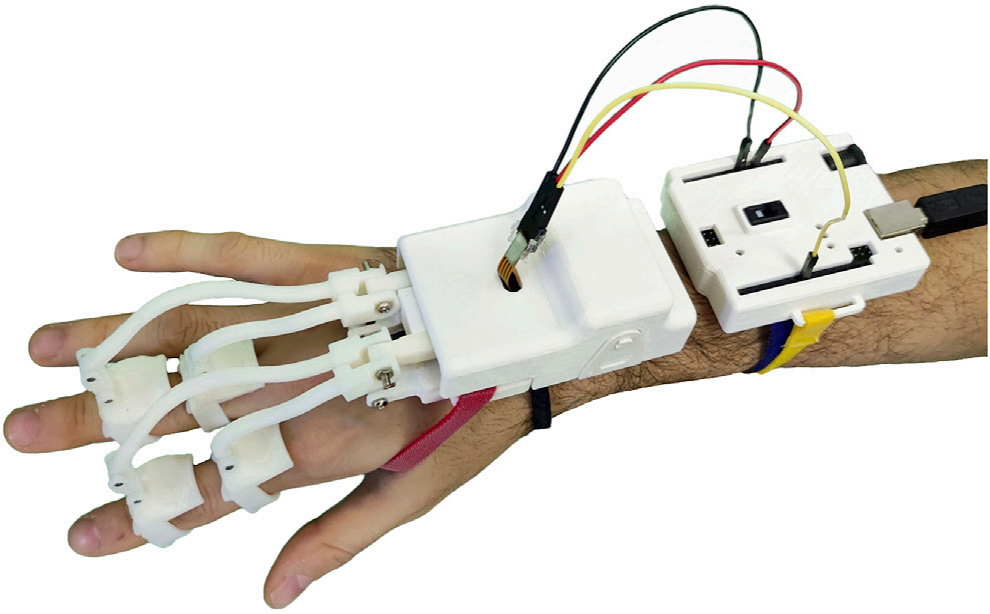
Prototype of the exoskeleton worn by the user.

In this study, we present the design and characterization of a modular hand finger exoskeleton, in which the number of actuators has been reduced by exploiting a gear-based miniaturized differential mechanism in order to limit the weight, complexity, and costs. One of the advantages of this kind of mechanism is that it is contained in a small box, while other solutions, such as tendon-based mechanisms, need a larger structure that could be uncomfortable for the user (Kang et al., 2015). The developed hand exoskeleton is shown in Figure 1. It is able to both flex and extend the two adjacent fingers in an independent way, with an actuator only. If one of the coupled fingers finds an obstacle in its movement, the other one can still continue its motion. The second contribution presented in this study is an improvement of the mechanical transmission between the actuators and the phalanges, with respect to the hand exoskeleton previously developed and summarized by Dragusanu et al., (2021), which is a part of a wider modular system that also includes the wrist. The device has been designed to be adopted in rehabilitation and telerehabilitation applications and to be used by the patient both in collaboration with the therapist or autonomously. Concerning the design and prototyping of the differential mechanism for the coupling of two adjacent finger modules, in particular, the study summarizes the following: 1) the mechanical, mechatronics, and manufacturing aspects of the mechanism, including its structural analysis, hardware ,and control description; 2) evaluating and comparing the proposed solution with respect the previous ones (Dragusanu et al., 2021) from the actuation point of view; and 3) presenting a working prototype and its functional testing in operative conditions.

The study is organized as follows. Section 2 summarizes the main requirements and criteria that guided the design illustrated in Section 3. Section 4 shows a prototype of the developed device and some tests performed to verify its functionality, and Section 5 provides a summary of the presented work and the directions of future investigation on this topic.

## 2 Background and Design Requirements

### 2.1 Kinematic Constraints: Summary of Human Hand Kinematics

The human hand has a quite complex kinematic structure, whose skeleton comprises 27 bones that can be divided into long and short bones. The carpus consists of eight short bones, while the remaining 19 long bones constitute the metacarpus, the four fingers, and the thumb. The bones lay the structural foundation for a complex mechanism with several DoFs (Degrees of Freedom). In the study by Tubiana, (1981) a 25 DoF model of the human hand is presented, while in the study by Jintae Lee and Kunii, (1995) the presented model has 30 DoFs. For the sake of simplicity, in this study, we refer to a simplified kinematic hand structure, in which we consider the carpus a unique rigid body while each finger is considered a planar kinematic chain, with two revolute joints in the proximal interphalangeal joint (PIP) and distal interphalangeal joint (DIP) and 2D hinges in the metacarpal joint (MCP). The thumb presents a different kinematics structure: a complete model of the thumb has two DoFs in the trapeziometacarpal joint (TC), two in the metacarpophalangeal joint, and one in the interphalangeal joint (IP). However, given the reduced range of motion of the abduction–adduction movement of thumb, the model can be reduced to a four DoF structure. Therefore, the reference hand model used in this study has 20 DoFs (Prattichizzo et al., 2013; Malvezzi et al., 2015).

The definition of the so-called postural synergies, the result of the psychophysics research presented by Santello et al. (1998), has also been exploited in robotics to manage the complexity of actuation systems. In this study, we used such a definition in the exoskeleton design to reduce the number of actuated DoFs without compromising the grip and manipulation capabilities of the hand. Postural synergies can be modeled as a mean for coordinating the large number of DoFs of the human hand, expressed with the joint variables $q\in R^{n_{q}}$ , with a reduced number of variables $z\in R^{n_{z}}$ , with *n*_*z*_ ≪ *n*_*q*_. The synergy constraint is typically expressed in terms of velocity as given by Prattichizzo et al., (2013):

$$\dot{q}=S\dot{z},$$ (1)

where $S\in R^{n_{q}\times n_{z}}$ is the so-called *synergy matrix*, $\dot{q}\in R^{n_{q}}$ is a vector containing joint angular velocities, and $\dot{z}$ represents synergy velocities. Columns of the synergy matrix $\hat{S}$ describe the so-called *postural synergies*. Postural synergies have been evaluated in the study by Santello et al., (1998) by processing a set of virtual grasp postures by means of the principal component analysis. A set of users were asked to shape their hands imagining to grasp an object from a quite large set (*N* = 57), and the corresponding postures were recorded using a CyberGlove system and the PCA was then applied to the obtained data. The results demonstrated that more than the 80*%* of the variance can be represented by the first two principal components, suggesting that out of the 25 DoFs of a human hand, only two or three combinations can be used to shape the hand for basic grasps used in everyday life. This simplification principle has been investigated in the design of underactuated robotic hands (Catalano et al., 2014).

In the studies by Gabiccini et al., (2011); Prattichizzo et al., (2013), the postural synergies defined by Santello et al., (1998) were adapted to the mathematical model of a human hand that was referred in those studies as the *paradigmatic* hand. The proposed model had 20 DoFs, and each finger had four DoFs. Since the data given in the study by Santello et al., (1998) were captured with a 15 DoF CyberGlove system, the obtained synergy matrix **S** dimensions were 20 × 15. Such a synergy matrix is available in SynGrasp Toolbox (Malvezzi et al., 2015) or can be evaluated with the data available in the study by Bianchi and Liarokapis, (2013).

Other studies available in the literature show that the complexity of human hand kinematics can be simplified considering joint coordination. For instance, Cobos et al. (2007) showed that the interphalangeal distal joint of each finger moves with motion equal to and equal to a fixed fraction of the interphalangeal proximal joint.

The concept of postural synergies has also been exploited in this study in the design of the hand exoskeleton to reduce the number of actuators. In particular, each module of the exoskeleton has been designed to flex and extend two fingers so that in each finger joint, rotation angles are coordinated according to the first postural synergy defined by Santello et al., (1998) available in the dataset presented by Bianchi and Liarokapis, (2013).

**FIGURE 2:**
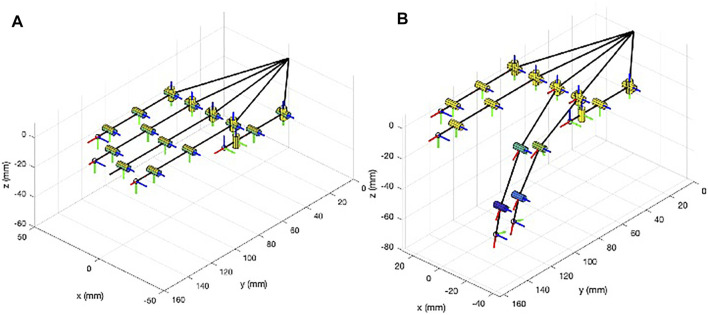
Schematic representation of the human hand 20 DoF model implemented in SynGrasp. **(A)** Hand in the reference open position. **(B)** Actuation of the first synergy for the index and middle flexion/extension motions.

In accordance with the abovementioned studies, the kinematic structure of the thumb is significantly different from the other finger ones, and it has not been exploited in this study. Furthermore, the device developed in this study is aimed at supporting the user in flexion/extension motion of the fingers, while adduction/abduction movements are not actuated. Consequently, the design of the exoskeleton modules will be synergy-based according to the following relationship:

$${\dot{q}}_{i}=S_{1,i}{\dot{z}}_{1,i},$$ (2)

$${\dot{q}}_{m}=S_{1,m}{\dot{z}}_{1,m},$$ (3)

where $q_{i}\in R^{2}$ and $q_{m}\in R^{2}$ are the velocity vectors relative to MCP (MetaCarpo-Interphalangeal) and PIP (Proximal-Interphalangeal) flexion/extension joints of the index and middle fingers, respectively. $S_{1,i}\in R^{2}$ and $S_{1,m}\in R^{2}$ are extracted from **S** matrix, in particular **S**_1,*i*_ contains the elements of the 1st column (first synergy) and 6th and 7th rows (MCP and PIP joints of the index finger), while **S**_1,*m*_ contains the elements of the 1st column and 10th and 11th rows (MCP and PIP joints of the middle finger) (Gabiccini et al., 2011; Prattichizzo et al., 2013). ${\dot{z}}_{1,i}$ and ${\dot{z}}_{1,m}$ are the synergy velocities for the index and middle fingers, respectively. If each finger is independently actuated, ${\dot{z}}_{1,i}$ and ${\dot{z}}_{1,m}$ are independent, while if the fingers are coupled by means of a differential mechanism, the following relationship between synergy velocities can be set:

$${\dot{z}}_{1}=\frac{{\dot{z}}_{1,i}+{\dot{z}}_{1,m}}{2},$$ (4)

in which ${\dot{z}}_{1}$ represents the synergy velocity actuated by the motor. Figure 2 reports the syngrasp simplified graphical representation of the human hand 20 DoF model in the reference open configuration and with the activation of the first postural synergy for the index and middle flexion motions.

### 2.2 Independent and Coupled Actuation of Fingers

Several actuating methods, active and passive, have been developed and implemented for hand exoskeletons. Among the active actuation methods, the most popular way of actuating hand exoskeletons is the one that exploits electric motors, that is, DC motors, brushless DC (BLDC) motors, servo motors, and linear actuators (du Plessis et al., 2021). Linear actuators are preferred in some applications because they can apply bidirectional forces and therefore can push or pull the mechanical linkage system to flex or extend the fingers (Dragusanu et al., 2021). Linear actuators that can be used in this type of applications have quite small dimensions so that one actuator per finger can be used. This kind of actuation has multiple advantages such as the possibility of making the exoskeleton’s fingers independent, allowing a precise control in finger movements.

Linear actuators have a finite stroke that limits the finger range of motion (ROM): the stroke that the actuator can realize is related to the ROM of the finger by means of exoskeleton kinematics relationships (Malvezzi et al., 2020). The linear actuator and the sizes of the linkages in the articulated transmission system have to be defined to allow the patient to fully flex and extend the finger, and this requirement could lead to a solution that is bulky and difficult to wear.

In this context, the miniaturized differential mechanism presented in this study allows to couple the actuation of two adjacent fingers, allowing the decrease of the number of actuators, reducing the weight and complexity of the system, and improving wearability. The differential mechanism does not rigidly connect the fingers but maintains them independently, so if one of the two branches of the differential is blocked, the other one can still move. It is worth noting that when blocking one of the fingers, the ROM of the other finger is doubled. With the same mechanical linkage system used to flex and extend the finger, by using the differential mechanism finger, the ROM can be increased with respect to the one that can be obtained, without the differential mechanism, limited by the actuator stroke.

Differential mechanisms are quite common in robotics, and in particular in robotic hands and fingers (Birglen and Gosselin, 2006). Planetary gear solutions have been presented for example by Fukaya et al., (2000); Birglen and Gosselin, (2003); Zappatore et al., (2017). In tendon-based systems, the moving pulley differential mechanisms can be used (Massa et al., 2002). In the study by Zappatore et al., (2017), a differential system based on gears is used for a novel architecture of the robotic hand, and the properties of differential mechanisms arranged in cascade *via* parallel or serial connections are studied. In the study by Baril et al., (2013), an underactuated anthropomorphic gripper for prosthetic applications is presented, in which a mechanical lever inside the palm is allowed to extend the grasping capabilities and improve the force transmission ratio of the gripper. This mechanism was further developed by Kontoudis et al., (2015), in which the differential mechanism included a set of locking buttons allowing the user to stop the motion of each finger.

### 2.3 Summary of the Main Exoskeleton Requirements

The design of the exoskeleton was performed in close collaboration with a potential user that continuously followed the development. The main requirements that guided the design of the exoskeleton for hand finger actuation, defined in accordance with the user, are reported in the following section.

• **Wearability**: the exoskeleton should be easily worn by the user, possibly without the need of external assistance.
• **Encumbrance**: the encumbrance of the exoskeleton should be as contained as much as possible, and the bottom surface of the fingers should not be constrained to allow the user to grasp and manipulate objects.
• **Number of actuated DoF**: for each couple of adjacent fingers (e.g. index/middle, ring/little), only one actuator is used, and the motion of MCP, PIP, and DIP of each finger should, therefore, be coupled by the mechanical structure of the exoskeleton transmission system, while the two fingers should be coupled through a differential system.
• **Type of motion**: the exoskeleton will actuate the flexion/extension motion of each finger only. The flexion/extension range for the MCP joint is [0, 90] deg.
• **Maximum force**: the developed exoskeleton will be able to apply a maximum equivalent force at the fingertip of 20 N both in the flexion and extension mode.

## 3 Design and Modeling

On the basis of the requirements described in Section 2, we designed and modeled the exoskeleton structure using the Autodesk Fusion 360. The device is designed to ensure that the movements of the exoskeleton match those of the human hand ones and do not constrain or overload hand joints.

### 3.1 Differential Mechanism

**FIGURE 3:**
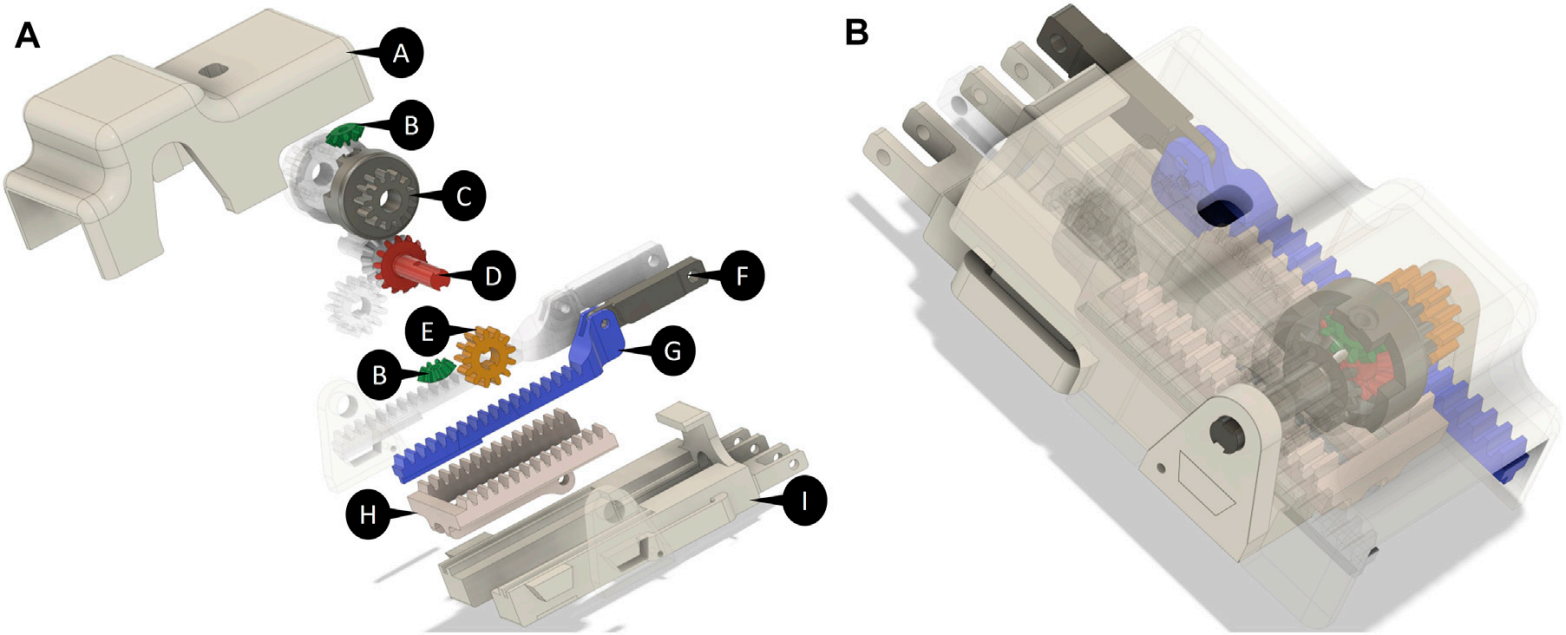
**(A)** Exploded CAD view of the differential mechanism. (A) Differential mechanism cover box (I) support for the linear actuator and for the differential mechanism parts. The differential mechanism comprises two satellite gears (B), two gears meshed with the shafts (D), two differential’s branches (G,F), a rack (H), and two spur gears (E) meshed with two crowns (C). **(B)** Assembled differential mechanism.

As previously introduced, a gear-based differential mechanism is used to couple the motion between two adjacent fingers. The CAD model of the differential mechanism is shown in Figure 3. The size of the gear-based differential is reduced as much as possible, and the resulting dimensions are a trade-off between wearability, weight, motion smoothness, mechanical resistance, and ease of manufacture with FDM (Fused Deposition Modeling) and SLA (Stereo-Lithography) technologies available in our laboratory. The gear-based differential mechanism is actuated by one linear actuator only, fully contained in the mechanism box.

The differential mechanism is an epicyclic gear with two DoFs, comprising two main parts:

• **The differential gearing** consists of four bevel gears, that is, two satellite (indicated with B in Figure 3A) gears and two gears joined to the shafts (D) that transmit the motion to the fingers of the exoskeleton by using the differential’s branches (G,F).
• **The carrier** is actuated by a rack (H), which is powered by a linear actuator placed in a support base pocket (I). It contains two sites where the satellite gears of the differential gearing are mounted.

The carrier is designed to allow the connection between the differential box and linear actuator. It is symmetrical and presents two spur gears on the side (E), joined to the crown (C in Figure 3A). The differential box contains the epicyclic gear. We choose a symmetric structure in order to homogenously distribute forces and torques, ensuring mechanical stability.

When the actuator is retracted, the hand is in the rest configuration (extended) pose. Instead, when the actuator is extended, it moves the rack, which is connected to the differential box that is free to rotate. If no obstacle is encountered in the movement, the satellites of the differential mechanism do not rotate with respect to their own axes, so the shafts rotate jointly with the crown, with the same angular velocity. The transmission gears that are keyed on the shafts actuate the two terminal racks connected to the fingers, allowing the flexion of the hand according to exoskeleton kinematics. If one finger finds an obstacle, the adjacent finger keeps moving with a double velocity, in accordance with the relation that describes the differential mechanism, that is:

$$\tau_{l,r}=\frac{\omega_{r}-\omega_{c}}{\omega_{l}-\omega_{c}}=-1\Rightarrow \omega_{c}=\frac{\omega_{l}+\omega_{r}}{2},$$ (5)

where *τ*_*l*,*r*_ is the transmission ratio of the differential mechanism, according to the Willis equation, *ω*_*r*_ and *ω*_*l*_ are, respectively, the angular velocities of the right and left shaft, and *ω*_*c*_ is the angular velocity of the carrier. Since the transmission ratio in a rack and pinion system is constant, the above mentioned relationship can be easily expressed as a function of linear actuator stroke *s* and differential output strokes for the index and middle fingers, indicated with *s*_*i*_ and *s*_*m*_, as follows:

$$s=\frac{s_{i}+s_{m}}{2}.$$ (6)

In this way, the device can move two adjacent fingers with an actuator only and a power source, with the advantage of keeping the motion of fingers independent.

### 3.2 Finger Actuation

As introduced in Section 2, the user’s comfort is an important requirement when we deal with wearable devices, especially if designed for rehabilitation. For this reason, the design of finger support is performed to increase the adaptability and wearability. The user should be able to wear/unwear the device easily and reasonably quickly, and the structure has to be easily adaptable to users with slightly different anthropometric measures.

It is worth noting that the developed transmission system is underactuated when it is worn on the finger, that is, the kinematic structure is “closed” by the finger; this feature allows better wearability and better adaptability to the user-specific finger dimensions (Dragusanu et al., 2021).

**FIGURE 4:**
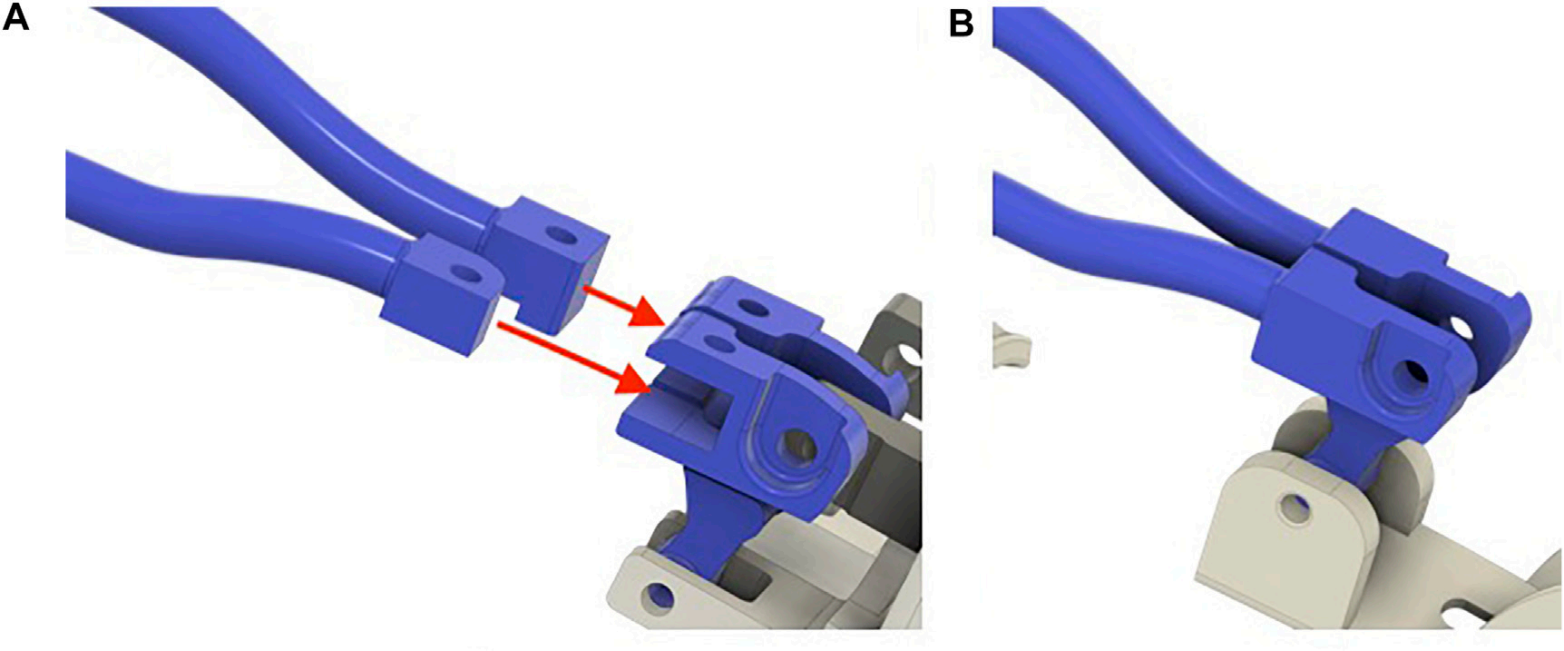
**(A)** New finger’s module socket. **(B)** Previous version of the finger’s module.

The finger module was previously presented and was developed to satisfy the abovementioned wearability and kinematic requirements. In the design proposed in this study, further improvements to the finger module have been implemented. In particular, the new version of the sockets, shown in Figure 4A, with respect to the version in Figure 4B, allows easy assembling/disassembling of the finger modules to/from the actuator/differential without using any tool.

**FIGURE 5:**
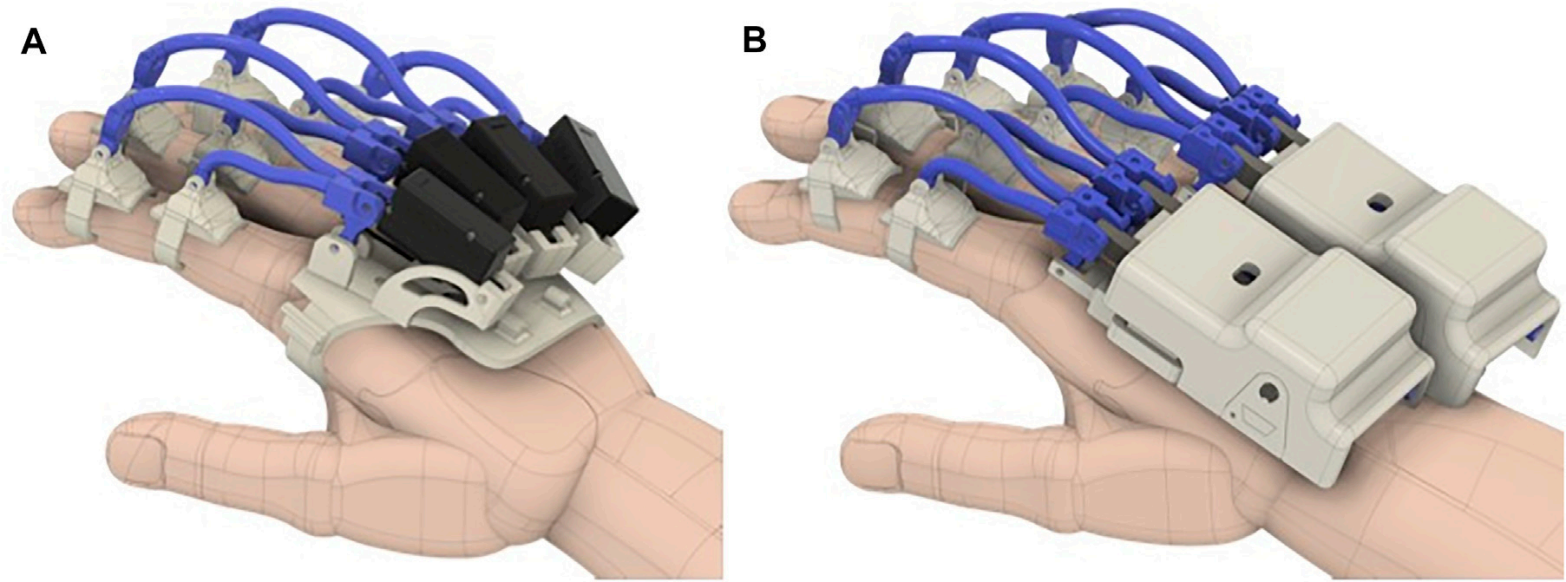
**(A)** CAD model of the previous version of the exoskeleton worn on the hand. **(B)** CAD model of the proposed exoskeleton worn on the hand.

The complete CAD model including the modules for the fingers and the differential mechanism is shown in Figure 5. In particular, 5A shows the solution previously introduced by Dragusanu et al., (2021), in which each finger is independently actuated, while 5B shows an upgrade of that version that further simplifies the assembly/deassembly of the device and further improves adaptability and wearability, in which fingers are coupled by two differentials and only two actuators are needed.

The mechanical transmission between differential outputs and finger phalanges has been developed to implement the concept of postural synergies introduced by the neuroscientific studies summarized in the previous section. In particular, the position and dimension of the exoskeleton linkages have been defined to reproduce the first postural synergy on each finger. At the same time, the differential mechanism allows maintaining the finger’s independent mechanism. In other words, for each finger, the transmission mechanism is designed to replicate the same coordination between the proximal and intermediate interphalangeal joints observed in the first postural synergy, but each finger can be independently moved.

**FIGURE 6:**
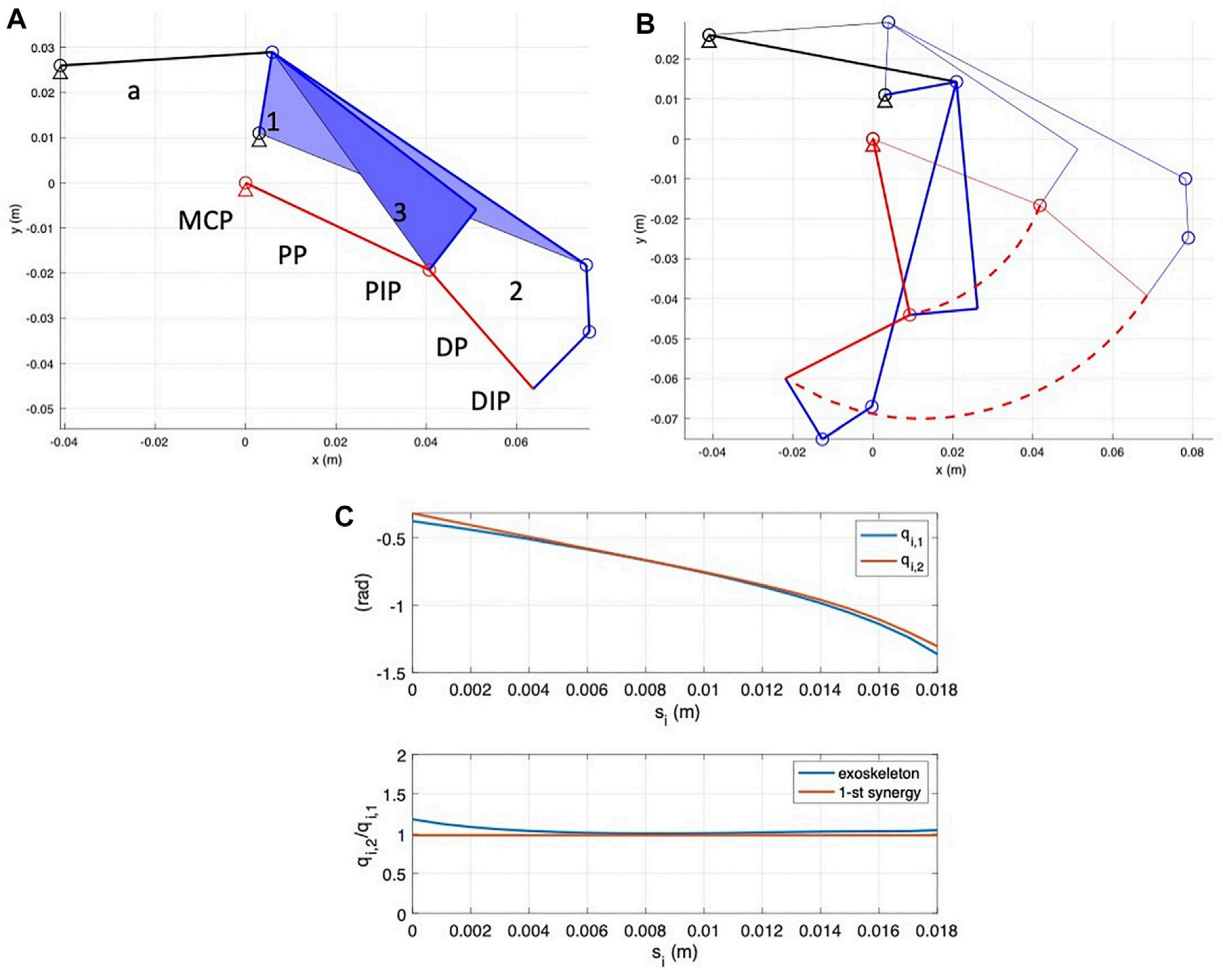
Kinematic scheme of the finger actuation system. **(A)** Kinematic scheme. The system comprises an actuator (a), two links for intermediate phalange actuation, connected to the finger in correspondence with the DIP joint (elements 1 and 2), and a link for proximal phalanx actuation, connected to the finger in correspondence of the PIP joint. Red links represent the simplified f2-DoF finger structure. **(B)** Kinematic simulation. Thin lines represent the reference initial configuration, thick continuous lines represent the final close configuration, and red dashed curves represent PIP and DIP point trajectories. **(C)** Results of kinematics analysis relative to the index finger module. Upper diagram: MPC and PIP joint rotation angles, *q*_1,*i*_ and *q*_2,*i*_ as a function of input (actuator or differential) stroke, indicated with *z*. Lower diagram: ratio between joint rotation angles; *q*_2,*i*_/*q*_1,*i*_ as a function of *z*, compared with the value obtained from the first postural synergy defined by Santello et al., (1998).

The finger actuation system has been developed so that when actuated, the flexion/extension motion follows, with a suitable approximation, the first postural synergy, as previously introduced. A kinematic analysis was, therefore, carried out to show how the trajectory of the exoskeleton matches with that of the human one (Cafolla and Carbone, 2014; Carbone et al., 2020). The simplified kinematic scheme of the transmission is represented in Figure 6A. From the kinematics point of view, the finger actuation mechanism comprises five rigid links: the actuator (a), represented by two rigid bodies, links 1 and 2, actuating the intermediate phalanx, and link 3 actuating the proximal phalanx. It is worth noting that element 1) represents the linear actuator for the device in which each finger is independently actuated and the differential output when the fingers are coupled. The rigid bodies are connected by five revolute (R) joints and a prismatic (P) joint (the actuator, not represented in the scheme), resulting in three DoFs. When the exoskeleton is worn on the finger, the finger kinematics structure *terminates* the mechanism. The resulting kinematic chain has seven bodies (the above-introduced links and finger proximal and intermediate phalanges), connected by 9 R-joints and one P-joint, with one residual DoF. A straightforward kinematic analysis allows estimating finger motion as a function of the stroke applied by the element (a).

Figure 6B shows system initial (thin lines) and final (thick lines) and PIP and DIP point trajectories. Figure 6C reports in the upper diagram the variation of index MPC and PIP angles, indicated by *q*_1,*i*_ and *q*_2,*i*_, respectively, as a function of element 1) stroke, indicated with *s*, while in the lower diagram, the ratio *q*_2,*i*_/*q*_1,*i*_. It is interesting to note that both *q*_1,*i*_ and *q*_2,*i*_ vary almost linearly with respect to *s*, and that therefore the ratio *q*_2,*i*_/*q*_1,*i*_ is almost constant. Such a ratio is very close to the value that can be obtained as *τ* = *S*_1,7_/*S*_1,6_ (reported as a red horizontal line in the diagram). This result confirms that the exoskeleton finger actuation couples the MPC and PIP joints so that the finger follows the first postural synergy when its closure motion is guided by the exoskeleton.

Similar results can be obtained for the middle finger. From the abovementioned results, it is possible to directly correlate by means of a linear relationship the synergy inputs for the single fingers *z*_1,*i*_ *z*_1,*m*_ to differential output strokes *s*_*i*_ and *s*_*m*_, that is, it is possible to find four coefficients, *a*_*i*_, *b*_*i*_, *a*_*m*_, and *b*_*m*_, such that

$$\begin{array}{ccc} s_{i} & = & a_{i}z_{1,i}+b_{i}, \end{array}$$ (7)

$$\begin{array}{ccc} s_{m} & = & a_{m}z_{1,m}+b_{m}, \end{array}$$ (8)

and finally, the input stroke provided by the linear actuator is related to *s*_*i*_ and *s*_*m*_ by the differential relationship introduced in the equation. (Anthonius Lim and Pranata, 2020).

Future improvements of this study will be devoted to analyze the sensitivity of these results with respect to model uncertainties and the user’s personal anthropometric parameters. Such studies will not only consider finger movements but also other more complex and coordinated tasks, for example, grasping. For this purpose, other types of simulation tools will be considered, as for instance the one proposed by Zappatore et al., (2019).

**FIGURE 7:**
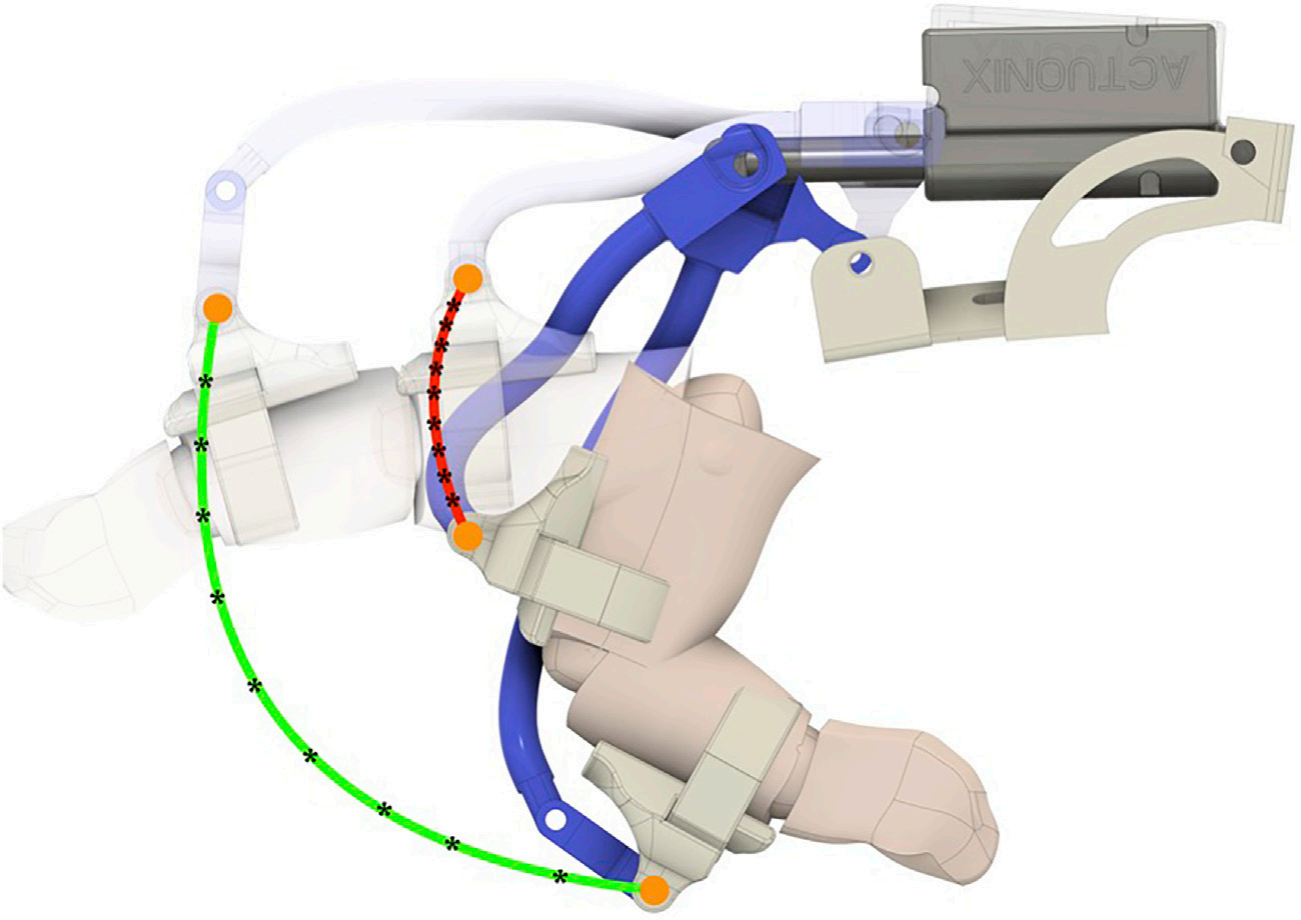
Multibody simulation of the finger exoskeleton worn on the index finger performed to verify that during closure motion, exoskeleton elements do not interfere with the finger.

The shape of the links has been designed to avoid interference with the finger during closure motion. Once the mechanism elements have been designed by means of a 3D CAD, a simple multibody configuration analysis was performed to verify that during the closure motion, the links do not interfere with subject phalanges. The results of this analysis are reported in Figure 7, in which the finger in the final closed configuration and the PIP and DIP point trajectories are reported.

### 3.3 Structural Analysis

In this section, we summarize the results of a set of basic structural analyses aimed at evaluating the structural resistance of the device and to select the materials to be used in the manufacturing phase. The structural analysis has been carried out on the finger actuation structure and on the differential mechanism components. Both the exoskeleton structures previously introduced have been considered.

The materials that have been considered for the mechanical structure of the finger and for the differential components were ABS, aluminum, and steel. For the actuator and the differential mechanism support, ABS has been considered. Finger supports for phalanges have been realized with ABS, while soft plastic has been used for the rings connecting the phalanges to the device. The studies have been realized with FEM analysis using 3D-CAD/CAE Autodesk^®^ Fusion 360 software.

### 3.3.1 Finger Actuation Structure

The two exoskeleton solutions previously introduced can be used with the same linear actuator or with the differential; they only differ in the mechanical structure that allows to assemble/disassemble the module, and then for all analyses, we neglected the linear actuator and focused on the mechanical structure study only.

**FIGURE 8:**
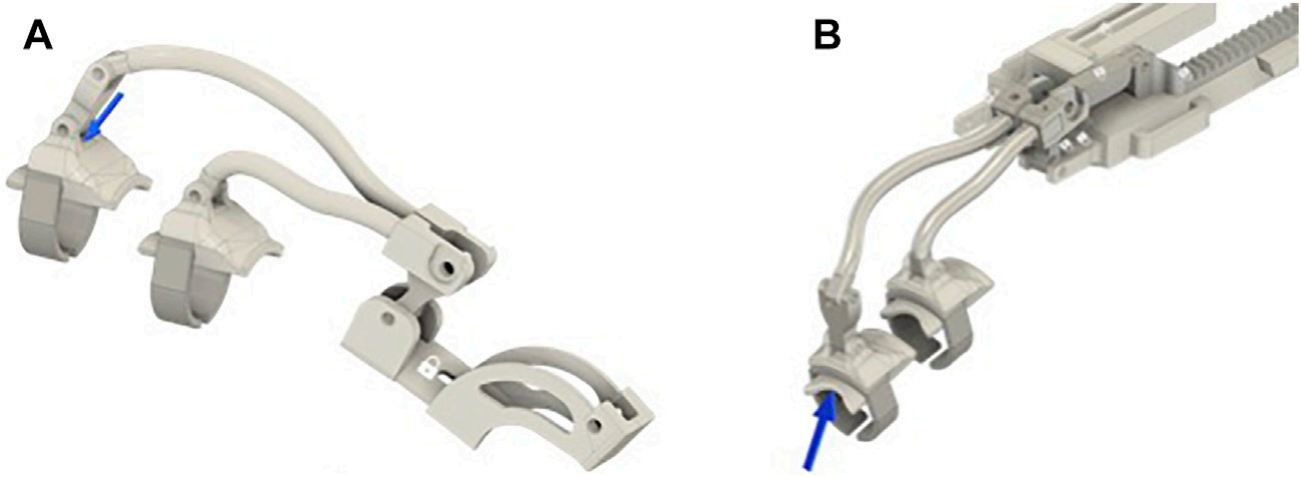
Models of the index module used for FEM analyses. In all the subfigures, the finger’s support is made with ABS while the ring comprises soft plastic material. **(A)** CAD model of the old index finer module solution, where the mechanism and the linear actuator’s support are made in ABS. **(B)** shows the CAD models of the new index finger module solution, where the finger mechanism and the differential mechanism support are made in aluminum. Blue arrows represent the applied forces, while the area signed with a white padlock indicates the constrained parts.

To make a comparison under similar conditions, we investigated the module designed for the index finger for both the solutions. The analysis was conducted considering different configurations, varying from the completely open to the completely closed one. The most critical loading condition corresponds to the completely open configuration. For the sake of conciseness, only the results relative to that configuration have been reported. In Figure 8, the two finger actuation structures with two of the three materials previously defined are shown. The figure also shows the points where the forces are applied and the constrained parts by using a blue arrow and a white padlock, respectively. Forces are applied to the extremity of the structure as this part is considered to be the area most involved in the hand rehabilitation process, aimed at reproducing the physiotherapist’s force during finger flexion/extension exercise. The magnitude of the applied force is 20 *N* both when the force points upward Figure 8A and when the force points downward (Figure 8B). The materials considered are ABS, steel, and aluminum.

**FIGURE 9:**
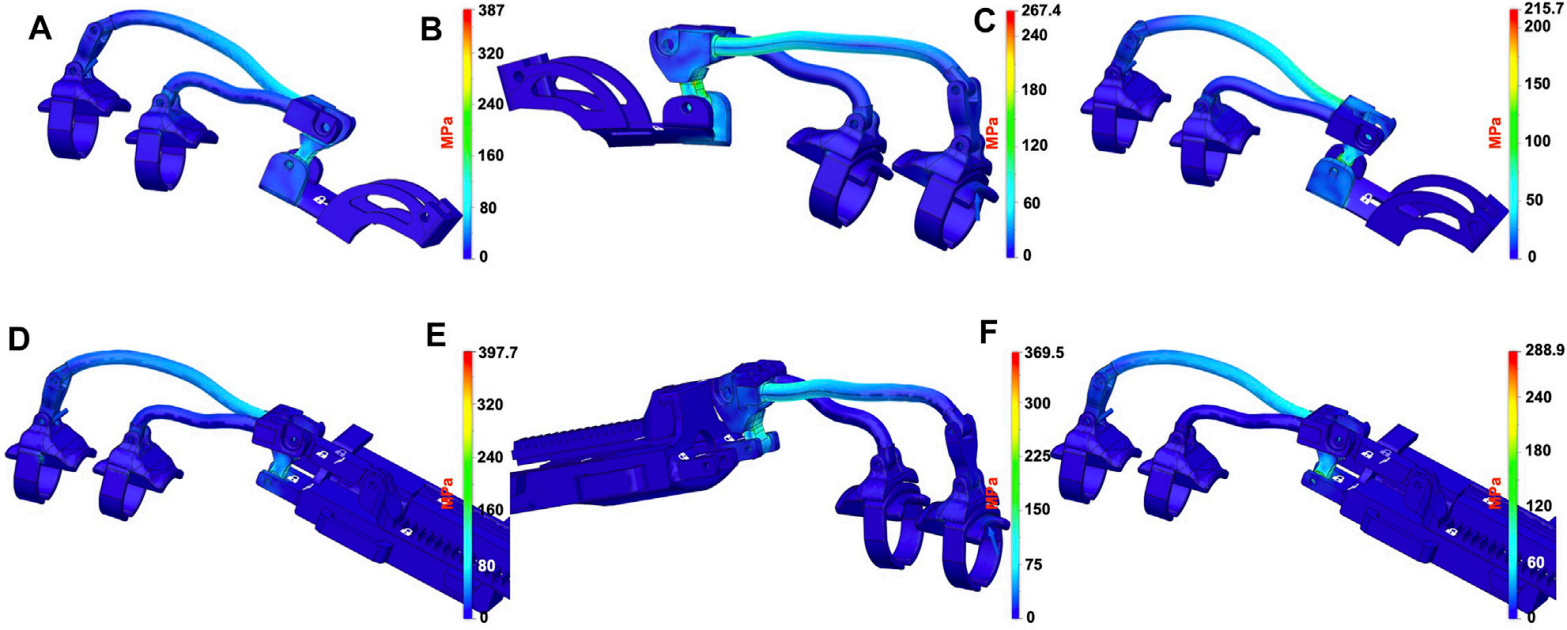
Results of the FEM structural analyses. Equivalent Von Mises stress distributions when a 20*N* magnitude force is applied on the distal part. The first row of subfigures reports the results for the previous finger module solution, when the materials used for the finger’s links are ABS **(A)**, aluminum, **(B)** and steel **(C)**. Subfigures **(D–F)** report the results for the new finger module solution when the materials used for the finger’s mechanism are ABS, aluminum, and steel, respectively.

**FIGURE 10:**
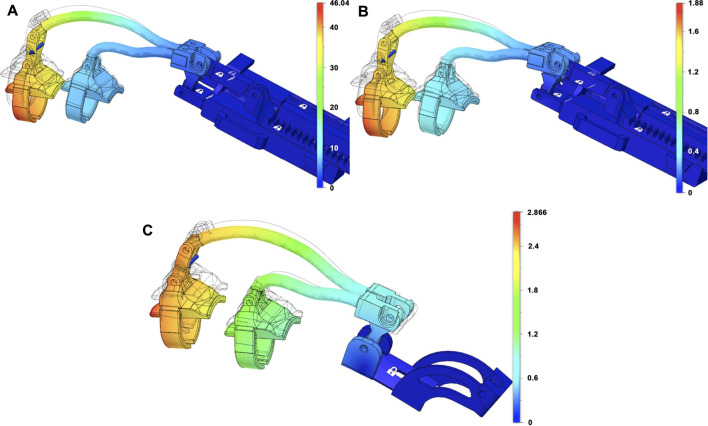
Results of the FEM structural analyses for the new **(A**,**B)** and old **(C)** index finger module in terms of displacements. The applied force has a magnitude of 20 N in all the cases, while the materials for the finger’s mechanism used is ABS in **(A)**, aluminum in **(B),** and steel in **(C)**.

A subset of the performed simulations is shown in Figures 9, 10, while Table 1 lists the main results of all analyses. It is worth observing that the maximum stress that can be sustained by a standard ABS is up to 60 *MPa*, and none of the models provides a compatible result with this constraint by applying a force of magnitude 20 *N*. The results show that none of the two solutions can be realized with this material unless the forces we want to apply are reduced or the structure is modified (increasing the thickness and, therefore, realizing a more bulky device). Regarding steel, the resistance coefficient is much higher, up to 500 *MPa*. In this case, the results show that in both the solutions, the maximum Von Mises equivalent stress is lower than that of the limit. Using aluminum, which has a maximum yield stress slightly lower than that of steel, the maximum equivalent Von Mises stress values are compatible with material properties. However, since the two materials have different Young’s modules, namely, 210 *GPa* for steel and 75 *GPa* for aluminum, the maximum displacements are different, even if their values are still limited. On the basis of these analyses, aluminum results in being the best material, among the analyzed ones, for the structural parts of the exoskeleton.

**TABLE 1:** Synthesis of the FEM analysis results in terms of stress and displacement with 20N of applied force magnitude.

	Force	ABS		Aluminum		Steel	
	N	Von Mises (MPa)	Displ (mm)	Von Mises (MPa)	Displ (mm)	Von Mises (MPa)	Displ (mm)
Old finger module solution	20	387.00	63.02	214.50	4.29	215.70	2.87
New finger module solution	20	397.70	46.04	255.10	1.88	288.90	0.70
Old finger module solution	-20	486.20	86.95	267.40	5.60	269.10	3.67
New finger module solution	-20	763.8	74.14	369.50	3.16	429.40	1.28

### 3.3.2 Differential Mechanism

**FIGURE 11:**
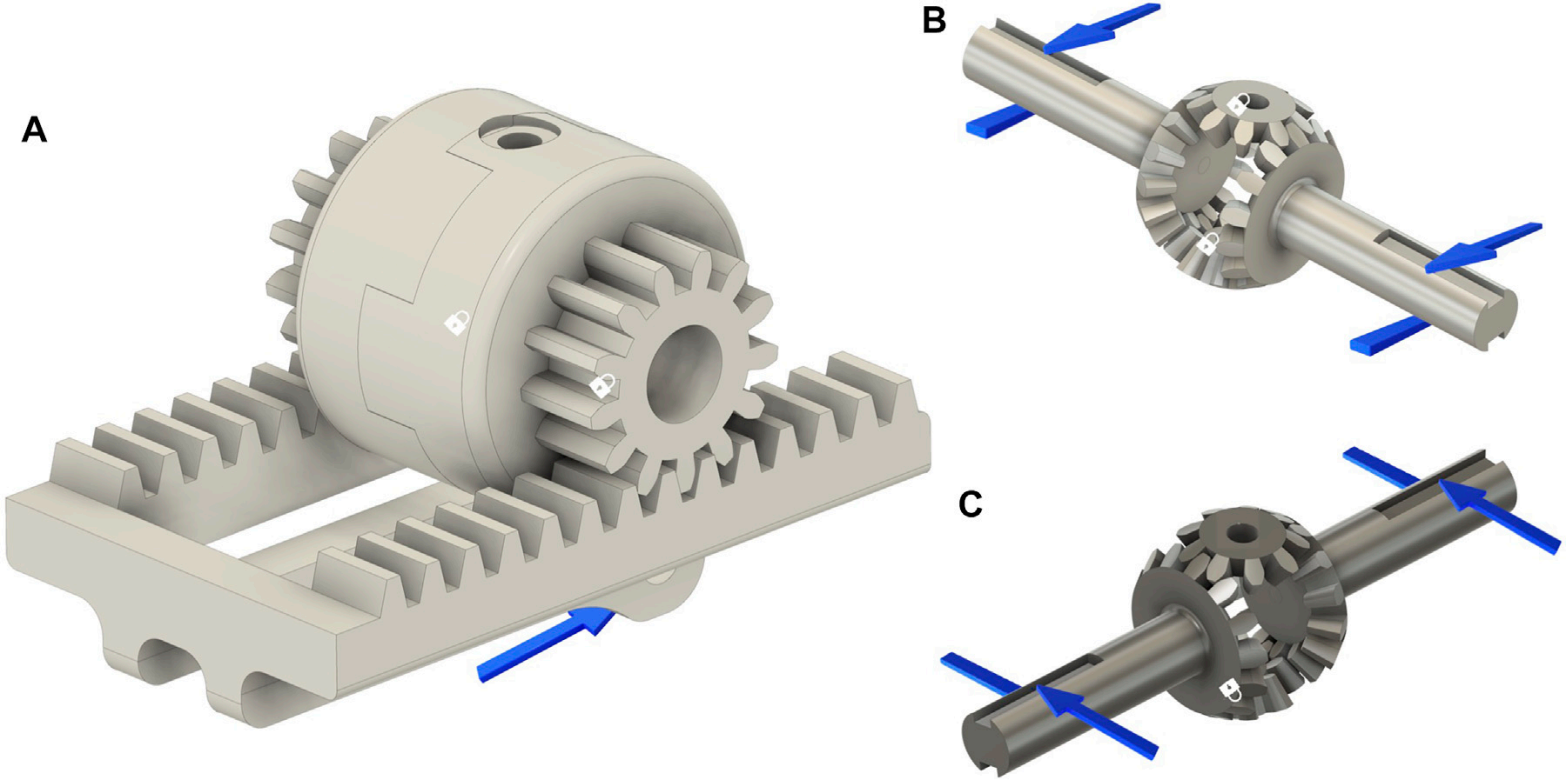
CAD model of the differential mechanism main parts used for FEM analyses. Blue arrows represent the applied forces, while the area signed with a white padlock indicates the constrained parts for the structural analyses. Subfigure **(A)** shows the carrier made in ABS material, while the other two subfigures show the differential gearing made in aluminum **(B)** and steel **(C)**.

We have studied the two main assembled parts that comprise the differential, that is, *the differential gearing* and *the carrier* to verify the overall stress and deformation level and define the best solution in terms of materials to be used for the realization of the proposed differential mechanism. In Figure 11, the two main parts of the differential mechanism with three different combinations of materials are shown. The padlock indicates the parts of the model in which constraints are set, while the blue arrows indicate the forces that are applied. In all the structural analyses, the magnitude and direction of the applied forces are evaluated by analyzing the statics of the whole device when a force with magnitude 20 *N* is applied on the finger. For the carrier, Figure 11A, the force is applied on the point in which the actuator connects to the rack and the constrained parts are inside the crown wheel. In order to study the critical points for the differential gears, forces and constraint are applied as shown in Figures 11B,C, In particular, the constraints are applied to the satellite gear bases, while the forces are applied to create a torque on the shafts.

**FIGURE 12:**
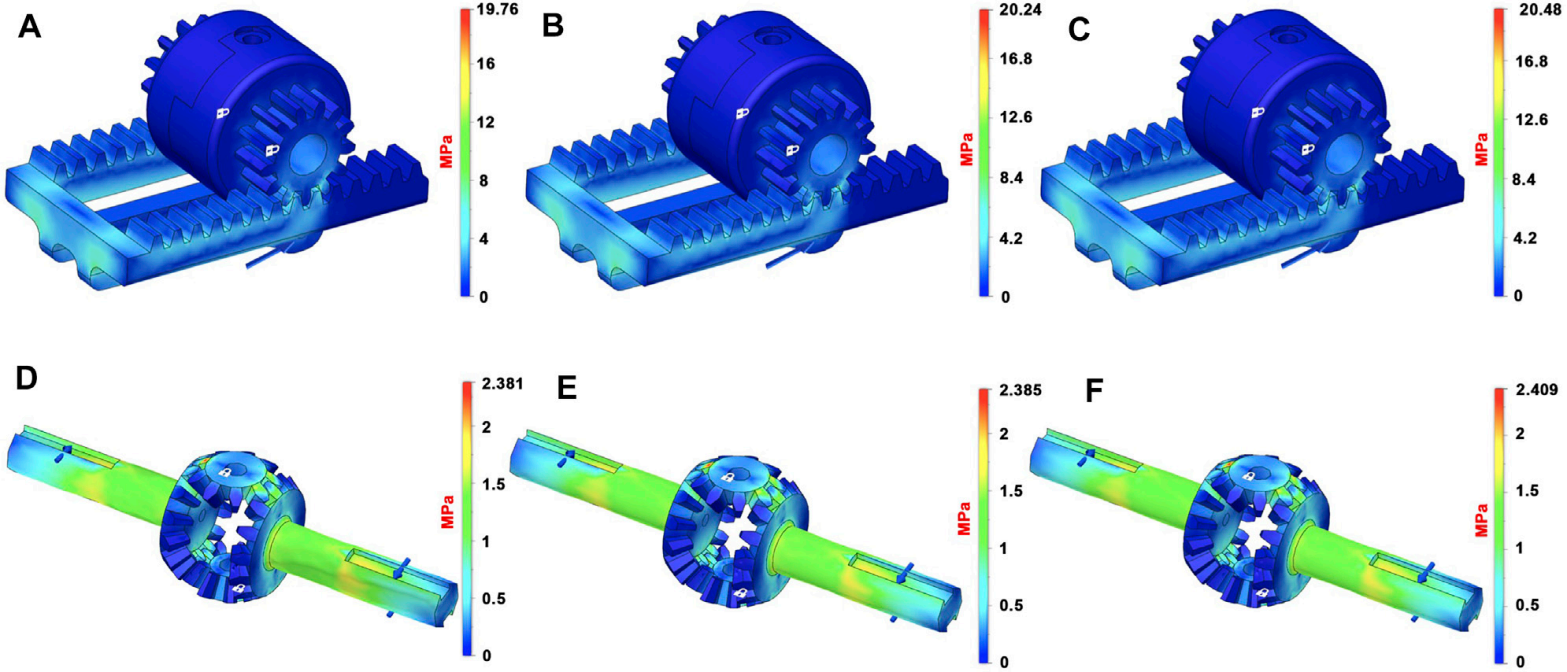
Results of the FEM structural analyses on the differential, equivalent Von Mises stress distributions. The first row of subfigures reports the results, for the carrier, of the FEM analysis when the materials used are ABS [**(A)**- max. Von Mises 19.76 MPa], aluminum [**(B)**- max. Von Mises 20.24 MPa], and steel [**(C)** - max. Von Mises 20.48 MPa]. Subfigures **(D–F)** report the results for the differential gearing when the materials are ABS (max. Von Mises 2.38 MPa), aluminum (max. Von Mises 2.39 MPa), and steel (max. Von Mises 2.41 MPa).

In Figure 12, simulation results are reported in terms of equivalent Von Mises stress, considering different materials. It can be observed that in all cases, the FEM analyses return acceptable Von Mises equivalent stress values. All results are under the maximum stress value of the analyzed materials. In this case, therefore, ABS could be a good solution because of its lightness, but on the other hand, this material presents some drawbacks in terms of wear and friction, which reduces the effectiveness. In addition, in this case, aluminum results in being the best solutions as it is lighter and more flexible than steel.

Table 2 summarizes the weights of both finger module solutions and the two main parts that comprise the proposed differential mechanism, based on the material used. From this table and from the above-presented results, the outcome is that the best solution, in terms of materials, is aluminum for the structural elements of the proposed device.

**TABLE 2:** Weights of the main parts of the exoskeleton.

	ABS weight (g)	Steel weight (g)	Aluminum weight (g)
Finger module	43.91	99.97	57.45
Differential	1.74	12.88	4.43
Carrier	9.07	67.13	23.09
Total	54.72	179.98	84.97

## 4 Prototype

This section presents the exoskeleton prototypes developed with the finger structures and the differential mechanism previously described. The exoskeleton is developed to be worn on the back of the hand, facilitating the user in the flexion and extension motions.

Even if from the analyses presented in the previous section, it results that the optimal choice for the material comprising structural parts is aluminum, in this phase of the development, the first prototype was realized in ABS using manufacturing techniques which are commonly available.

In particular, the FDM technique (Fused Deposition Modeling) was used to manufacture all the components. For the design and development of the exoskeleton, we followed a specific procedure, as outlined by Cafolla et al., (2016), that can be briefly summarized in the following: CAD modeling with Autodesk Fusion 360, conversion of the CAD model to STL, and transferring the STL file to the 3D-Printer. For the physical realization, we used the 3D-printer *Stratasys F123*. The mechanical components were printed using the GrabCAD Print. Components indicated with *B*, *C*, *D*, *E*, *G*, and *H* in Figure 3 were printed with a slicing height of 0.18 mm, while the other components, which did not need a high surface precision, were printed with a slicing height of 0.25 mm.

**FIGURE 13:**
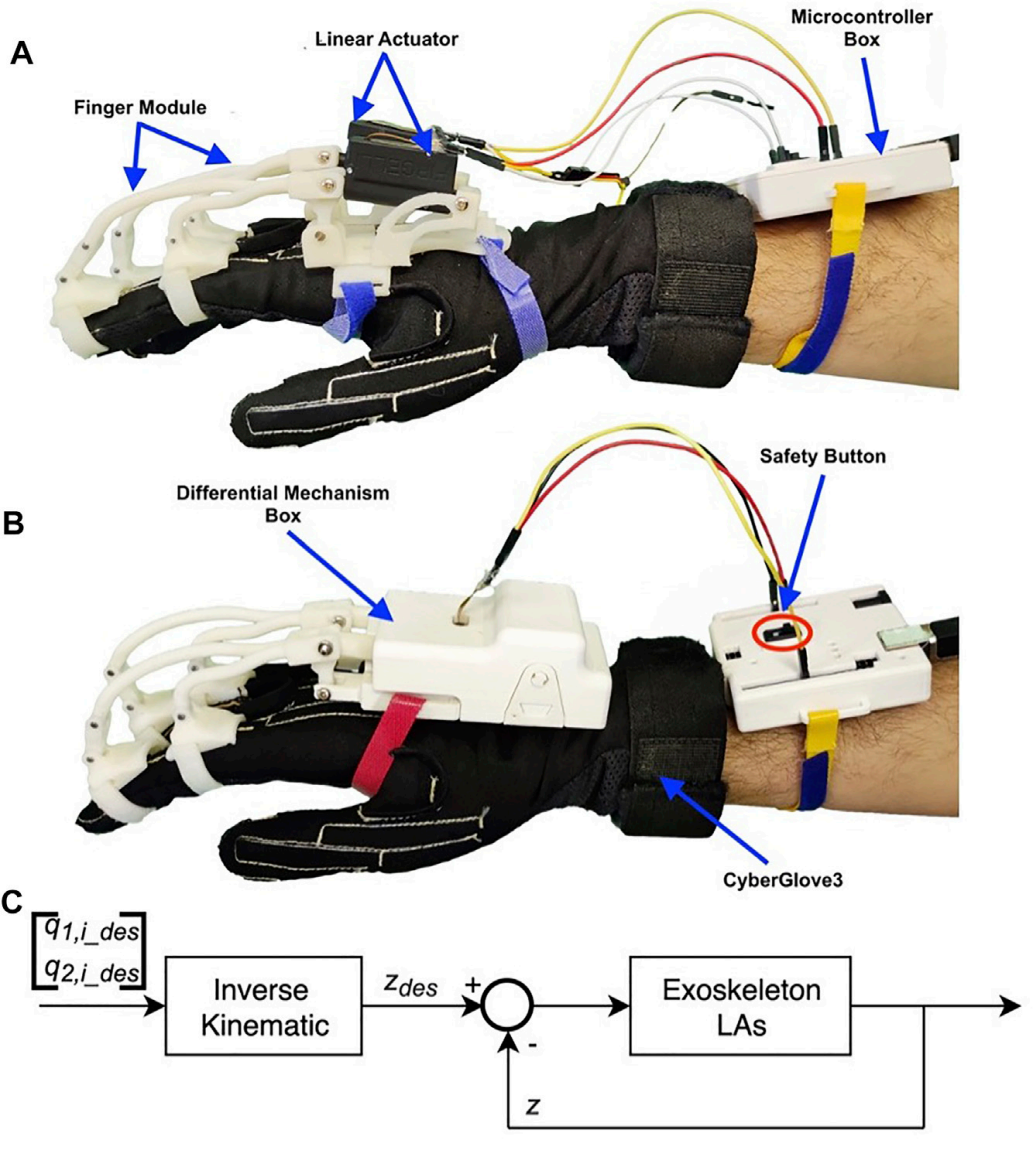
Experimental setup for the validation phases using CyberGlove3. Subfigure **(A)** shows two previous finger modules, while subfigure **(B)** shows the proposed device, both worn by the user. **(C)** Exoskeleton position control scheme.

The device is actuated by a linear actuator that is found between fingers, blocked in a pocket. The used linear actuator is the Actuonix PQ-12. This actuator has low weight, a stroke of 20 *mm* that can exert a force suitable to allow the user to apply a maximum equivalent force at the fingertip of 20 *N* both in the flexion and extension modes. The force is generated by a DC motor that is connected with a worm gear, which makes the motion of the shaft possible. The main features of the linear actuator are summarized in Table 3. To control the motion of the linear actuator and validate the prototype, an Arduino Uno is used. The actuator position is controlled according to the scheme presented in Figure 13B. For a given desired posture, expressed in terms of the index and middle joint rotation angles **q**_*des*_, an inverse kinematic procedure similar to the one presented by Mulatto et al., (2012) is used to estimate the corresponding synergy value *z*_*des*_ that is furthermore converted in the actuator desired stroke *s*_*des*_. A standard PID controller is then used to control the linear actuator. Control and electronic components are worn on the forearm with an armband as shown in Figure 13C. The device communicates *via* Bluetooth with a PC graphical user interface that allows managing and monitoring exoskeleton motions (Dragusanu et al., 2020b).

**TABLE 3:** Main characteristics of the Actuonix PQ-12 linear actuator.

Technical Features	
Max. force (lifted)	45 N
Stroke	20 mm
Mass	15 g
Feedback potentiometer	5 kΩ
Stall current	550 mA @ 6V
Max duty cycle	20%
Max speed (no load)	15 mm/s

Moreover, the hand exoskeleton is a module of a more complex device, which is designed for helping people with injuries of the upper limb (Dragusanu et al., 2020b). This device includes a module specially designed for the rehabilitation of the wrist that involves the implementation of a transmission based on tendons to actuate the wrist motions. It is made of thermoplastic material, such as to be adapted to the user’s specific needs.

### 4.1 Experimental Validation

The prototypes of the exoskeleton presented in this study has been tested and compared with its previous versions. Six subjects aged 24–30 years, four males and two females, wore a CyberGlove3 (CyberGlove Systems. Inc., US), a commercial hand tracking system with 22 joint angle sensors, and then they wore the previous version of the exoskeleton described by Dragusanu et al., (2021), as shown in Figure 13A and with each of these, they completed five opening and closing repetitions. They repeated the task wearing the new exoskeleton and in addition, each participant completed a further cycle of five openings and closings with one side of the differential mechanism mechanically blocked by the experimenter. Each subject took part in the entire experimental validation, gave her/his written informed consent to participate, and was able to interrupt participation at any time during experiments. The experiment protocols followed the Declaration of Helsinki, and there was no risk of harmful effects on the subjects’ health.

Through the CyberGlove3, the joint values of the metacarpal and the proximal of the index and the middle of each participant were recorded. The range of the movement performed with the two versions of the exoskeleton was then evaluated as the absolute difference of the mean values of each valley with the mean values of each peak immediately following.

**FIGURE 14:**
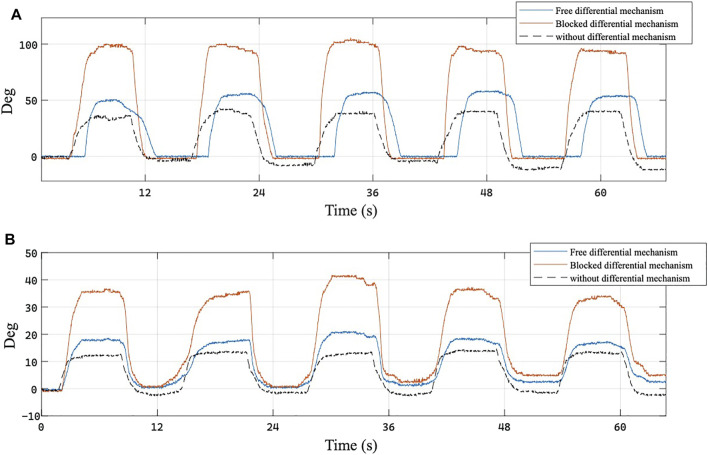
Joint rotation angles of the **(A)** metacarpo-phalangeal (MCP) and **(B)** proximal interphalangeal (PIP) joint of the middle finger recorded by CyberGlove during a single repetition of the physiotherapy task. Blue curve represents the joint angle obtained with the exoskeleton equipped with the differential mechanism, red curve represents the rotation of the same joint when the other finger is blocked, and black curve represents joint rotation obtained with the previous version of the exoskeleton.

The experimental results show that the new exoskeleton in free conditions extends the ROM of the wearing hand by about 10%, and just less than 100% in the case of one side of the differential mechanism has been mechanically blocked. It has been observed that the metacarpal joints make a movement wider than 14,02 ± 6.84% with the free differential mechanism and 90.02 ± 8.34% with the blocked mechanism, while the proximal joints extend their mobility by 13.21 ± 6.67% in the first case and 69,61 ± 27.03% in the other. One of the experimental trials is reported in Figure 14.

### 4.2 Using the Device With the Commercial Tracking System

**FIGURE 15:**
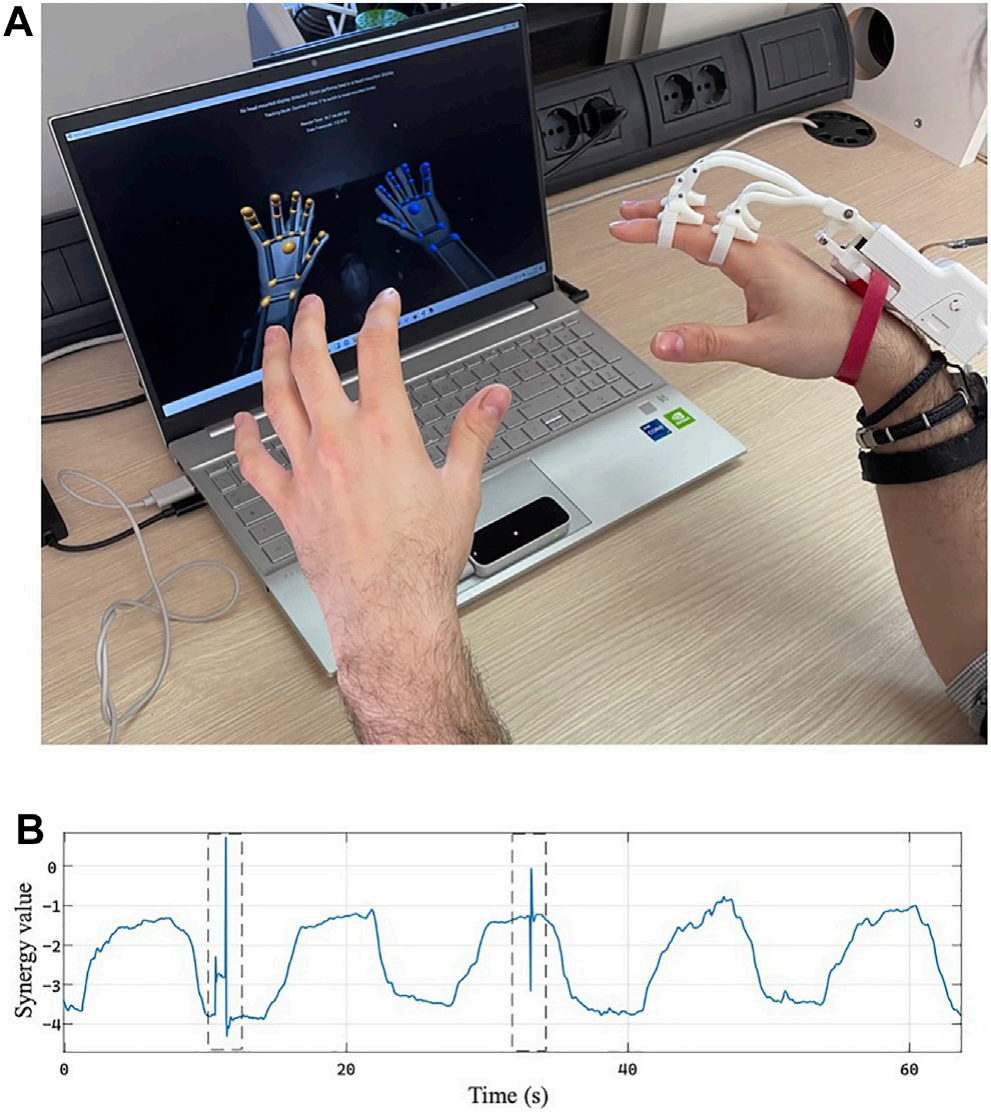
Use of the proposed exoskeleton with the LeapMotion Controller. **(A)** Experimental setup. **(B)** First synergy activation value *z* estimated during the acquisition of the LeapMotion Controller during the physiotherapy task. The dotted rectangles highlight the time interval in which the tracking system loses the hand in the frame.

Further tests were conducted to demonstrate the usability of the proposed exoskeleton with a widely diffused and affordable commercial optical tracking system. This aspect is important to let the device be used in real rehabilitation contexts beyond research laboratories, in which a sophisticated tracking system cannot be adopted due to high costs and complexity. Specifically, the possibility of using the exoskeleton with the LeapMotion Controller (UltraLeap.Inc., US) was tested, as is shown in Figure 15A. The metrics used to evaluate if the device can be used with this type of connection system is the number of average disconnections during a bimanual task: results obtained with and without the exoskeleton were compared.

Six subjects aged between 24 and 30 years, three males and three females, participated in the experimentation campaign; they were asked to complete three test cycle comprising five opening and closing movements performed simultaneously with both hands, the left one without the exoskeleton while the right one assisted by the exoskeleton. In addition, for this other experimental campaign, each subject took part in the entire experimental validation, gave her/his written informed consent to participate, and was able to interrupt the participation at any time during experiments. The experiment protocols followed the Declaration of Helsinki, and there was no risk of harmful effects on the subjects’ health.

In particular, the subjects involved in the experiments were volunteer collaborators of our laboratory, with extensive experience in testing new wearable devices. General precautions and safety measures were implemented as far as possible to prevent risks for all the people involved in device development, including 1) supervision of all experiments with the developed devices by at least one technically skilled supervisor who is able to interrupt the device function immediately in case of technical dysfunction or discomfort of the subjects; 2) software tools to detect malfunction and automatically inactivate the devices if necessary; and 3) physical measures to detach the participants from the devices in case the contact forces exceed a certain limit. The subjects involved in the project were not to be exposed to unnecessary risk. Additional safety procedures were applied to comply with the COVID-19 pandemic–related issues. No personal data relative to the subjects were collected during the tests.

The movements were constantly acquired through LeapMotion and mapped joint-to-joint in the movements of two virtual hand avatars on the screen placed in front of the participants.

The number of hand-lost for both hands and the status of the tracking system during the experimentation were recorded, and the obtained results are reported in Table 4.

**TABLE 4:** Number of times the hand assisted by the exoskeleton was lost by the LeapMotion Controller tracking system for each individual trial depending on the quality of brightness and cleanliness of the sensor reported by the diagnostics software: G means good; B means bad.

Trial	1	2	3	4	5	6	7	8	9	10	11	12	13	14	15	16	17	18
Smudge status	B	B	G	G	G	G	G	G	G	G	G	G	G	B	B	G	G	G
Lighting status	G	G	G	G	G	G	B	B	B	G	G	G	B	B	B	B	G	G
Exoskeleton	2	1	1	0	0	2	2	3	1	2	1	0	2	4	3	2	0	0
Free hand	0	1	0	0	0	1	2	2	1	0	0	0	1	2	2	2	0	0

The results show an inevitable difference in the number of hand-lost between the free hand and the hand assisted by the exoskeleton; the free hand is always tracked better or equally than the hand assisted by the exoskeleton. The average number of hand-lost in a time interval of about 60 s is equal to 1.44 ± 1.19 for the assisted hand and 0.78 ± 0.88 for the free hand. However, as can be seen from the graph shown in Figure 15B, the number of disconnections of the assisted hand and the duration of the same with respect to the length of the task is overall not excessive. These results allow asserting that the tracking system and assistive exoskeleton can be jointly used in rehabilitation exercises. The outcome of these tests, therefore, allows us to conclude that the physiotherapy exercises performed with the presented exoskeleton can be recorded and remotely monitored through commercial systems with low economic impact and wide diffusion.

## 5 Conclusion

In this study, we presented an actuation system for a hand finger exoskeleton in which the motion of two adjacent fingers is coupled by a differential mechanism. The developed gear-based differential mechanism homogenously distributes the actuator’s force, while keeping the motion of the coupled fingers independent so that if one of them is blocked because of contact with an obstacle, the other one is still able to move.

The reduction of the number of actuators is a significant advance for the device that is lighter, less complex, and with lower energy consumption, therefore with a longer battery life cycle. In addition, the updated transmission system allows obtaining an ROM of the phalanges about 10% wider than that the previous version, with the same actuator stroke. The device has also been tested in terms of compatibility with a simple and widely used tracking system with low economic impact, such as the LeapMotion Controller, to demonstrate that with the presented version of the exoskeleton, it is not only possible to carry out physiotherapy tasks but also to record and monitor them remotely, thus allowing to expand the rehabilitation possibilities and opportunities for patients, doctors, and physiotherapists. The compatibility with widely diffused tracking systems also allows increasing the acceptance of physiotherapy allowing patients to interact with virtual worlds and scenarios making the therapy an interactive, playful, and/or competitive act with themselves or with digital avatars and artificial intelligence.

The first impressions received from the user involved in the design phase turn out to be positive and optimistic, hence considering the device lighter and more practical with respect to other devices and hence considering natural movement, and overall the device was evaluated as comfortable both for wearability and functionality. A still open challenge is represented by the mechanical resistance; it has been verified from numerical simulations that ABS is not a suitable solution for the most involved components in terms of maximum stress and friction; however, this type of material has been used in the first prototyping phase because manufacturing technologies for this material are widely available. Future developments of this study will include the realization of prototypes with higher resistance materials (e.g. aluminum).

## Data Availability

The original contributions presented in the study are included in the article/Supplementary Material, further inquiries can be directed to the corresponding author.

## References

[B1] Anthonius Lim M. Pranata R. (2020). Letter to the Editor Regarding ‘the Challenging Battle of Mankind against Covid-19 Outbreak: Is This Global International Biological Catastrophe the Beginning of a new era?’–is Telehealth the Future of Orthopaedic and Rehabilitation in post-covid-19 Era?”. 10.1177/2309499020947840 32869701

[B2] Baril M. Laliberté T. Gosselin C. Routhier F. (2013). On the Design of a Mechanically Programmable Underactuated Anthropomorphic Prosthetic Gripper. J. Mech. Des. 135 (12), 121008. 10.1115/1.4025493

[B3] Bartlett N. W. Lyau V. Raiford W. A. Holland D. Gafford J. B. Ellis T. D. (2015). A Soft Robotic Orthosis for Wrist Rehabilitation. J. Med. Devices 9 (3). 10.1115/1.4030554

[B4] Bataller A. Cabrera J. A. Clavijo M. Castillo J. J. (2016). Evolutionary Synthesis of Mechanisms Applied to the Design of an Exoskeleton for finger Rehabilitation. Mechanism Machine Theor. 105, 31–43. 10.1016/j.mechmachtheory.2016.06.022

[B5] Bianchi M. Liarokapis M. V. (2013). “Handcorpus, a New Open-Access Repository for Sharing Experimental Data and Results on Human and Artificial Hands,” in IEEE World Haptics Conference (WHC),

[B6] Bianchi M. Cempini M. Conti R. Meli E. Ridolfi A. Vitiello N. (2018). Design of a Series Elastic Transmission for Hand Exoskeletons. Mechatronics 51, 8–18. 10.1016/j.mechatronics.2018.02.010

[B7] Birglen L. Gosselin C. M. (2003). “On the Force Capability of Underactuated Fingers,” in 2003 IEEE International Conference on Robotics and Automation (Cat. No. 03CH37422), Taipei, Taiwan, 14-19 Sept. 2003 (IEEE), 1139–1145.

[B8] Birglen L. Gosselin C. M. (2006). Force Analysis of Connected Differential Mechanisms: Application to Grasping. Int. J. Robotics Res. 25 (10), 1033–1046. 10.1177/0278364906068942

[B9] Cafolla D. Carbone G. (2014). “A Study of Feasibility of a Human finger Exoskeleton,” in Service Orientation in Holonic and Multi-Agent Manufacturing and Robotics (Springer), 355–364. 10.1007/978-3-319-04735-5_24

[B10] Cafolla D. Ceccarelli M. Wang M. Carbone G. (2016). 3d Printing for Feasibility Check of Mechanism Design. Int. J. Mech. Control. 17 (1), 3–12.

[B11] Carbone G. Gerding E. C. Corves B. Cafolla D. Russo M. Ceccarelli M. (2020). Design of a Two-Dofs Driving Mechanism for a Motion-Assisted finger Exoskeleton. Appl. Sci. 10 (7), 2619. 10.3390/app10072619

[B12] Catalano M. G. Grioli G. Farnioli E. Serio A. Piazza C. Bicchi A. (2014). Adaptive Synergies for the Design and Control of the Pisa/iit Softhand. Int. J. Robotics Res. 33 (5), 768–782. 10.1177/0278364913518998

[B13] Cobos S. Ferre M. Sánchez-Urán M. A. Ortego J. (2007). Constraints for Realistic Hand Manipulation. Proc. Presence, 369–370.

[B14] Díaz I. Catalan J. M. Badesa F. J. Justo X. Lledo L. D. Ugartemendia A. (2018). Development of a Robotic Device for post-stroke home Tele-Rehabilitation. Adv. Mech. Eng. 10 (1), 1687814017752302. 10.1177/1687814017752302

[B15] Dragusanu M. Baldi T. L. Iqbal Z. Prattichizzo D. Malvezzi M., (2020) Development of a Wearable Exoskeleton for Hand/wrist Rehabilitation and Training.

[B16] Dragusanu M. Lisini Baldi T. Iqbal Z. Prattichizzo D. Malvezzi M. (2020), “Design, Development and Control of a Tendon-Actuated Exoskeleton for Wrist Rehabilitation and Training,” in Proc. IEEE Int. Conf. on Robotics and Automation, Paris, France, 31 May-31 Aug. 2020, . 10.1109/icra40945.2020.9197013

[B17] Dragusanu M. Iqbal Z. Prattichizzo D. Malvezzi M. (2021). “Synthesis and Design of a Modular Hand Exoskeleton for Rehabilitation and Training,” in ASME 2021 IMECE, International Mechanical Engineering Congress and Exposition, November 1-5 2021.

[B18] du Plessis T. Djouani K. Oosthuizen C. (2021). A Review of Active Hand Exoskeletons for Rehabilitation and Assistance. Robotics 10 (1), 40. 10.3390/robotics10010040

[B19] Fukaya N. Toyama S. Asfour T. Dillmann R. (2000). “Design of the Tuat/karlsruhe Humanoid Hand,” in Proceedings. 2000 IEEE/RSJ International Conference on Intelligent Robots and Systems (IROS 2000)(Cat. No. 00CH37113), Takamatsu, Japan, 31 Oct.-5 Nov. 2000 (IEEE), 1754–1759.3

[B20] Gabiccini M. Bicchi A. Prattichizzo D. Malvezzi M. (2011). On the Role of Hand Synergies in the Optimal Choice of Grasping Forces. Autonomous Robots 31 (2), 235–252. 10.1007/s10514-011-9244-1

[B21] Gopura R. A. R. C. Bandara D. S. V. Kiguchi K. Mann G. K. I. (2016). Developments in Hardware Systems of Active Upper-Limb Exoskeleton Robots: A Review. Robotics Autonomous Syst. 75, 203–220. 10.1016/j.robot.2015.10.001

[B22] Gorgey A. S. Sumrell R. Goetz L. L. (2019). Exoskeletal Assisted Rehabilitation after Spinal Cord Injury. Atlas of Orthoses and Assistive Devices, 440–447. 10.1016/b978-0-323-48323-0.00044-5

[B23] Gupta A. O'Malley M. K. Patoglu V. Burgar C. (2008). Design, Control and Performance of Ricewrist: a Force Feedback Wrist Exoskeleton for Rehabilitation and Training. Int. J. Robotics Res. 27 (2), 233–251. 10.1177/0278364907084261

[B24] Hesse S. Schmidt H. Werner C. Bardeleben A. (2003). Upper and Lower Extremity Robotic Devices for Rehabilitation and for Studying Motor Control. Curr. Opin. Neurol. 16 (6), 705–710. 10.1097/00019052-200312000-00010 14624080

[B25] Jintae Lee J. Kunii T. L. (1995). Model-based Analysis of Hand Posture. IEEE Comput. Grap. Appl. 15 (5), 77–86. 10.1109/38.403831

[B26] Jo I. Park Y. Lee J. Bae J. (2019). A Portable and spring-guided Hand Exoskeleton for Exercising Flexion/extension of the Fingers. Mechanism Machine Theor. 135, 176–191. 10.1016/j.mechmachtheory.2019.02.004

[B27] Kang B. B. In H. Sin M. Cho K.-J. (2015). Exo-glove: A Wearable Robot for the Hand with a Soft Tendon Routing System. IEEE Robotics Automation Mag. 22 (1), 97–105.

[B28] Kontoudis G. P. Liarokapis M. V. Zisimatos A. G. Mavrogiannis C. I. Kyriakopoulos K. J. (2015). “Open-source, Anthropomorphic, Underactuated Robot Hands with a Selectively Lockable Differential Mechanism: Towards Affordable Prostheses,” in 2015 IEEE/RSJ international conference on intelligent robots and systems (IROS), Hamburg, Germany, 28 Sept.-2 Oct. 2015 (IEEE), 5857–5862. 10.1109/iros.2015.7354209

[B29] Krebs H. I. Volpe B. T. Williams D. Celestino J. Charles S. K. Lynch D. (2007). Robot-aided Neurorehabilitation: a Robot for Wrist Rehabilitation. IEEE Trans. Neural Syst. Rehabil. Eng. 15 (3), 327–335. 10.1109/tnsre.2007.903899 17894265 PMC2733849

[B30] Lum P. S. Burgar C. G. Shor P. C. Majmundar M. Van der Loos M. (2002). Robot-assisted Movement Training Compared with Conventional Therapy Techniques for the Rehabilitation of Upper-Limb Motor Function after Stroke. Arch. Phys. Med. Rehabil. 83 (7), 952–959. 10.1053/apmr.2001.33101 12098155

[B31] Malvezzi M. Gioioso G. Salvietti G. Prattichizzo D. (2015). Syngrasp: A Matlab Toolbox for Underactuated and Compliant Hands. IEEE Robot. Automat. Mag. 22 (4), 52–68. 10.1109/mra.2015.2408772

[B32] Malvezzi M. Baldi T. L. Villani A. Ciccarese F. Prattichizzo D. (2020). “Design, Development, and Preliminary Evaluation of a Highly Wearable Exoskeleton,” in 2020 29th IEEE International Conference on Robot and Human Interactive Communication (RO-MAN), Naples, Italy, 31 Aug.-4 Sept. 2020 (IEEE), 1055–1062. 10.1109/ro-man47096.2020.9223604

[B33] Marconi D. Baldoni A. McKinney Z. Cempini M. Crea S. Vitiello N. (2019). A Novel Hand Exoskeleton with Series Elastic Actuation for Modulated Torque Transfer. Mechatronics 61, 69–82. 10.1016/j.mechatronics.2019.06.001

[B34] Martinez-Martin E. Cazorla M. (2019). Rehabilitation Technology: Assistance from Hospital to home. Comput. intelligence Neurosci. 2019. 10.1155/2019/1431509 PMC6589308 31281333

[B35] Massa B. Roccella S. Carrozza M. C. Dario P. (2002). “Design and Development of an Underactuated Prosthetic Hand,” in Proceedings 2002 IEEE international conference on robotics and automation (Cat. No. 02CH37292), Washington, DC, 11-15 May 2002 (IEEE), 3374–3379.4

[B36] Moreno-SanJuan V. Cisnal A. Fraile J.-C. Pérez-Turiel J. de-la-Fuente E. (2021). Design and Characterization of a Lightweight Underactuated Raca Hand Exoskeleton for Neurorehabilitation. Robotics Autonomous Syst. 143, 103828. 10.1016/j.robot.2021.103828

[B37] Mulatto S. Formaglio A. Malvezzi M. Prattichizzo D. (2012). Using Postural Synergies to Animate a Low-Dimensional Hand Avatar in Haptic Simulation. IEEE Trans. Haptics 6 (1), 106–116. 10.1109/TOH.2012.13 24808272

[B38] Peretti A. Amenta F. Tayebati S. K. Nittari G. Mahdi S. S. (2017). Telerehabilitation: Review of the State-Of-The-Art and Areas of Application. JMIR Rehabil. Assist. Technol. 4 (2), e7. 10.2196/rehab.7511 28733271 PMC5544892

[B39] Popov D. Gaponov I. Ryu J.-H. (2016). Portable Exoskeleton Glove with Soft Structure for Hand Assistance in Activities of Daily Living. IEEE/ASME Trans. Mechatronics 22 (2), 865–875.

[B40] Prattichizzo D. Malvezzi M. Gabiccini M. Bicchi A. (2013). On Motion and Force Controllability of Precision Grasps with Hands Actuated by Soft Synergies. IEEE TRANSACTIONS ROBOTICS 29 (6). 10.1109/tro.2013.2273849

[B41] Rahman M. A. Al-Jumaily A. (2012). Design and Development of a Hand Exoskeleton for Rehabilitation Following Stroke. Proced. Eng. 41, 1028–1034. 10.1016/j.proeng.2012.07.279

[B42] Randazzo L. Iturrate I. Perdikis S. Millán J. d. R. (2017). A Wearable Hand Exoskeleton for Activities of Daily Living and Neurorehabilitation. IEEE Robotics Automation Lett. 3 (1), 500–507.

[B43] Rehmat N. Zuo J. Meng W. Liu Q. Xie S. Q. Liang H. (2018). Upper Limb Rehabilitation Using Robotic Exoskeleton Systems: a Systematic Review. Int. J. Intell. Robot Appl. 2 (3), 283–295. 10.1007/s41315-018-0064-8

[B44] Santello M. Flanders M. Soechting J. F. (1998). Postural Hand Synergies for Tool Use. J. Neurosci. 18 (23). 10.1523/JNEUROSCI.18-23-10105.1998 PMC6793309 9822764

[B45] Shahid T. Gouwanda D. Nurzaman S. G. Gopalai A. A. (2018). Moving toward Soft Robotics: A Decade Review of the Design of Hand Exoskeletons. Biomimetics 3 (3), 17. 10.3390/biomimetics3030017 PMC6352684 31105239

[B46] Tubiana R. (1981). Architecture and Functions of the Hand. Oxford, England: Oxford University, 19–93.

[B47] Wang D. Meng Q. Meng Q. Li X. Yu H. (2018). Design and Development of a Portable Exoskeleton for Hand Rehabilitation. IEEE Trans. Neural Syst. Rehabil. Eng. 26 (12), 2376–2386. 10.1109/tnsre.2018.2878778 30387735

[B48] World Health Organization (2016). World Health Statistics 2016: Monitoring Health for the SDGs Sustainable Development Goals. Geneva, Switzerland: World Health Organization.

[B49] World Health Organization (2021). World Health Statistics 2021: Monitoring Health for the SDGs Sustainable Development Goals. Geneva, Switzerland: World Health Organization.

[B50] Zappatore G. A. Reina G. Messina A. (2017). Analysis of a Highly Underactuated Robotic Hand. Int. J. Mech. Control. 18 (4), 17–23.

[B51] Zappatore G. Reina G. Messina A. (2019). A Toolbox for the Analysis of the Grasp Stability of Underactuated Fingers. Robotics 8 (2), 26. 10.3390/robotics8020026

[B52] Zhang F. Hua L. Fu Y. Chen H. Wang S. (2014). Design and Development of a Hand Exoskeleton for Rehabilitation of Hand Injuries. Mechanism Machine Theor. 73, 103–116. 10.1016/j.mechmachtheory.2013.10.015

